# Supplementary material for: Fangchinoline induces gallbladder cancer cell apoptosis by suppressing PI3K/Akt/XIAP axis
Source: PLoS One. 2022 Apr 21;17(4):e0266738. doi: 10.1371/journal.pone.0266738 (PMC9022853; doi:10.1371/journal.pone.0266738)
Supplement: S1 File — (ZIP) [file pone.0266738.s001.zip › S1 File/Supporting information/Western blot/S1_raw_images.pdf]

GBC-SD

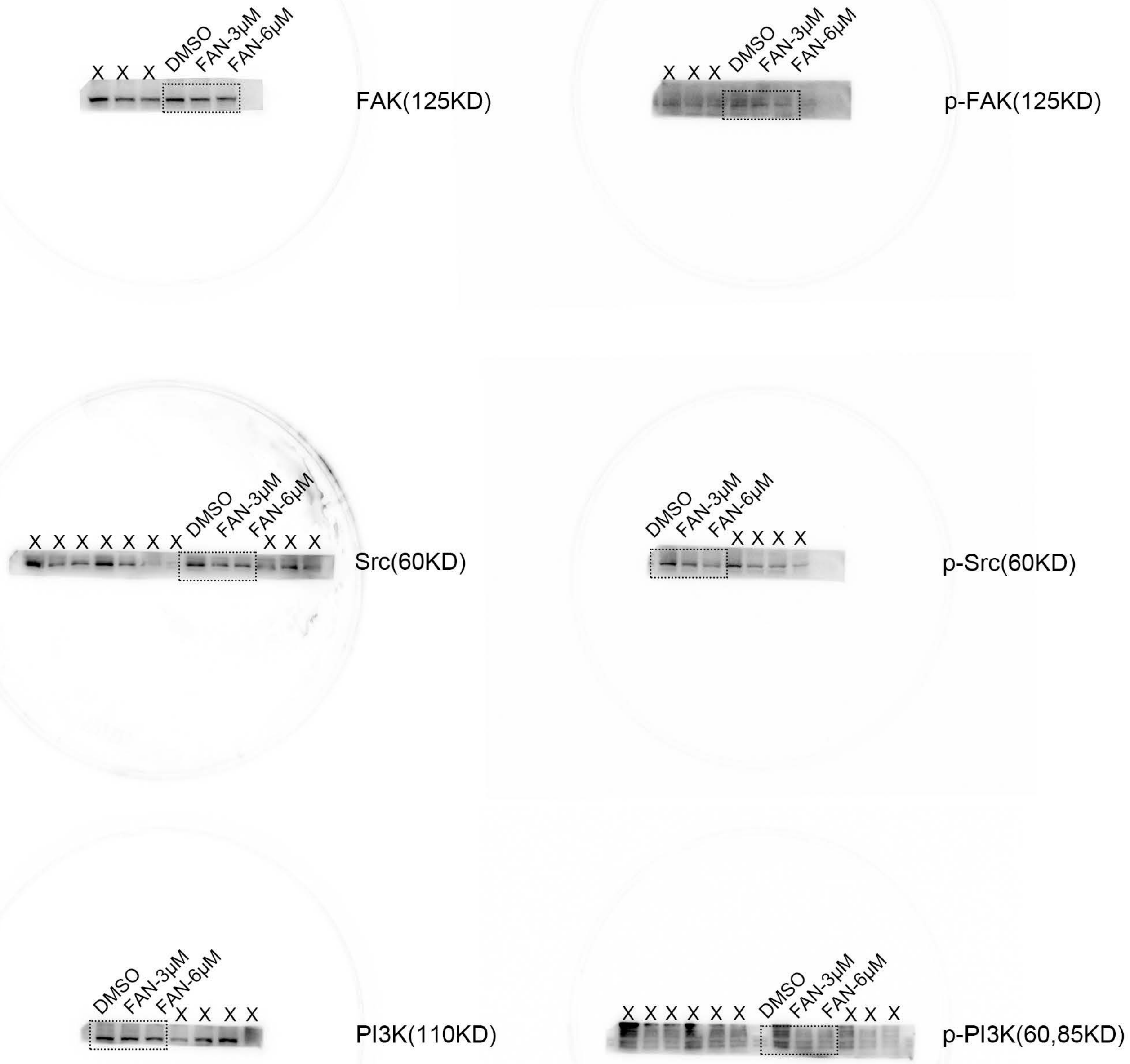
X X X DMSO FAN-3μM FAN-6μM
FAK(125KD)
X X X DMSO FAN-3μM FAN-6μM
p-FAK(125KD)
X X X X X X X DMSO FAN-3μM FAN-6μM X X X
Src(60KD)
DMSO FAN-3μM FAN-6μM X X X X
p-Src(60KD)
DMSO FAN-3μM FAN-6μM X X X X
PI3K(110KD)
X X X X X X DMSO FAN-3μM FAN-6μM X X X
p-PI3K(60,85KD)

GBC-SD

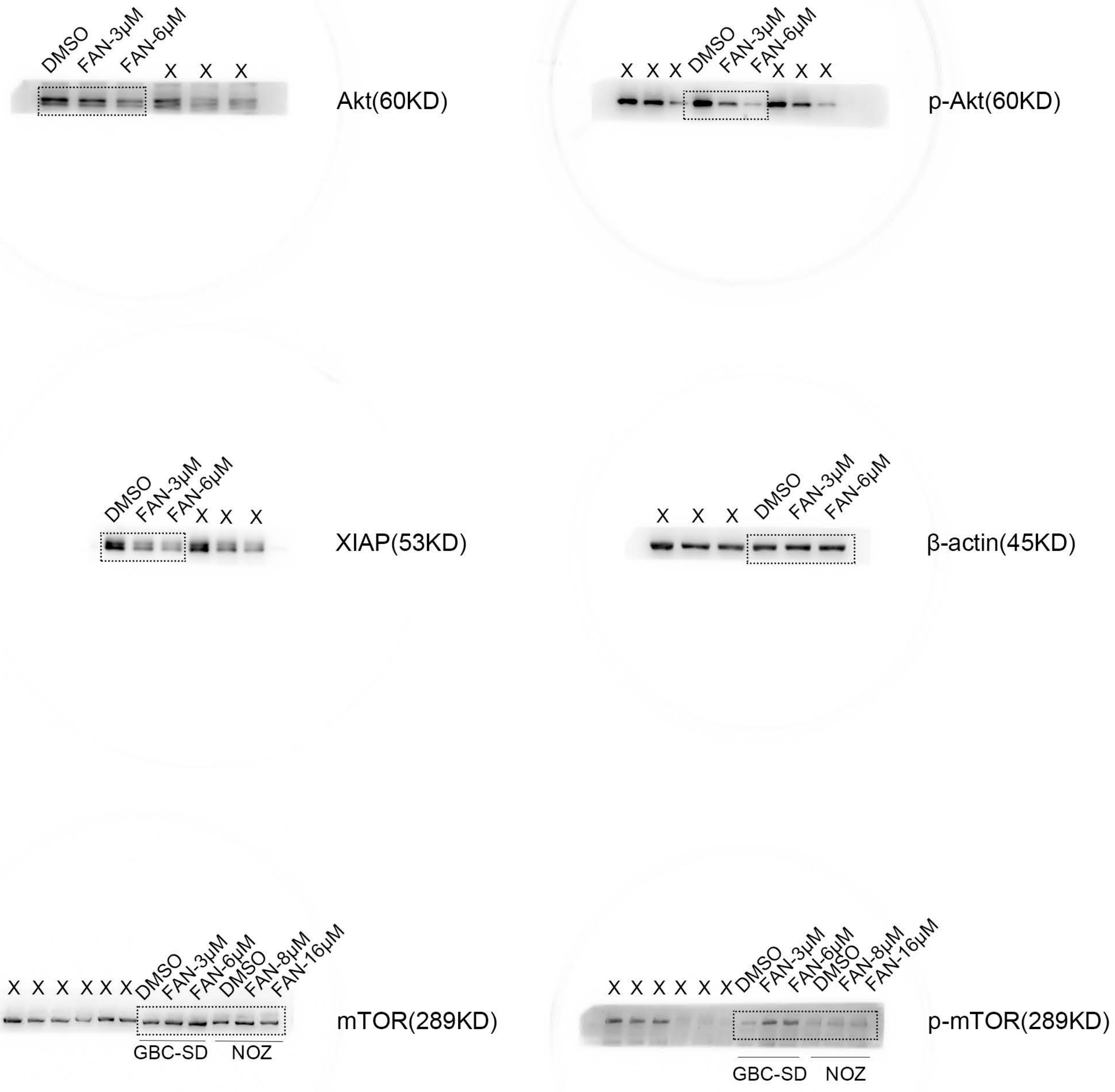
DMSO
FAN-3μM
FAN-6μM
X X X
Akt(60KD)
X X X
DMSO
FAN-3μM
FAN-6μM
X X X
p-Akt(60KD)
DMSO
FAN-3μM
FAN-6μM
X X X
XIAP(53KD)
X X X
DMSO
FAN-3μM
FAN-6μM
β-actin(45KD)
X X X X X X
DMSO
FAN-3μM
FAN-6μM
DMSO
FAN-8μM
FAN-16μM
mTOR(289KD)
GBC-SD
NOZ
X X X X X X
DMSO
FAN-3μM
FAN-6μM
DMSO
FAN-8μM
FAN-16μM
p-mTOR(289KD)
GBC-SD
NOZ

NOZ

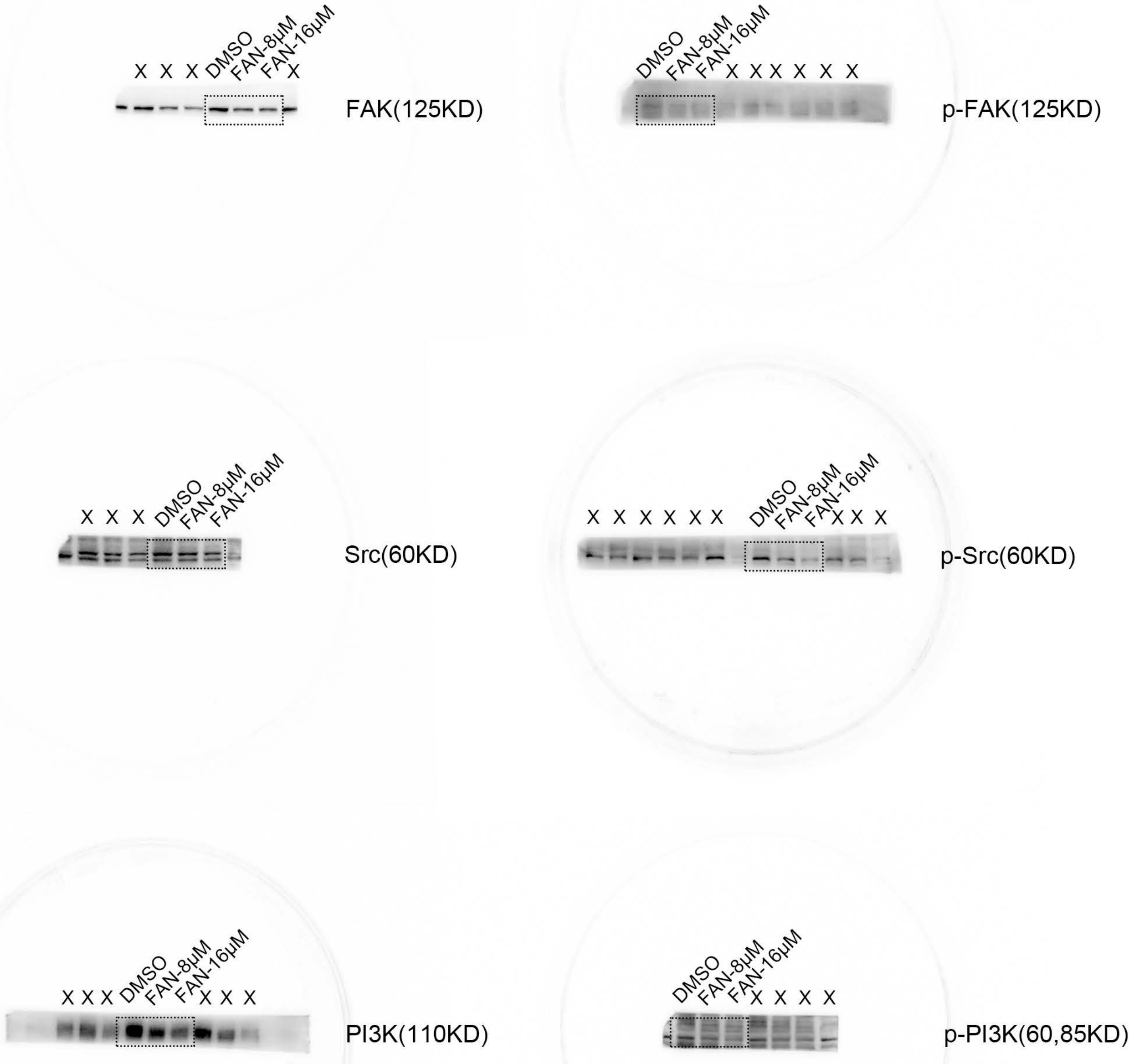
X X X DMSO FAN-8μM FAN-16μM X
FAK(125KD)
DMSO FAN-8μM FAN-16μM X X X X X X
p-FAK(125KD)
X X X DMSO FAN-8μM FAN-16μM
Src(60KD)
X X X X X X DMSO FAN-8μM FAN-16μM X X X
p-Src(60KD)
X X X DMSO FAN-8μM FAN-16μM X X X
PI3K(110KD)
DMSO FAN-8μM FAN-16μM X X X X
p-PI3K(60,85KD)

NOZ

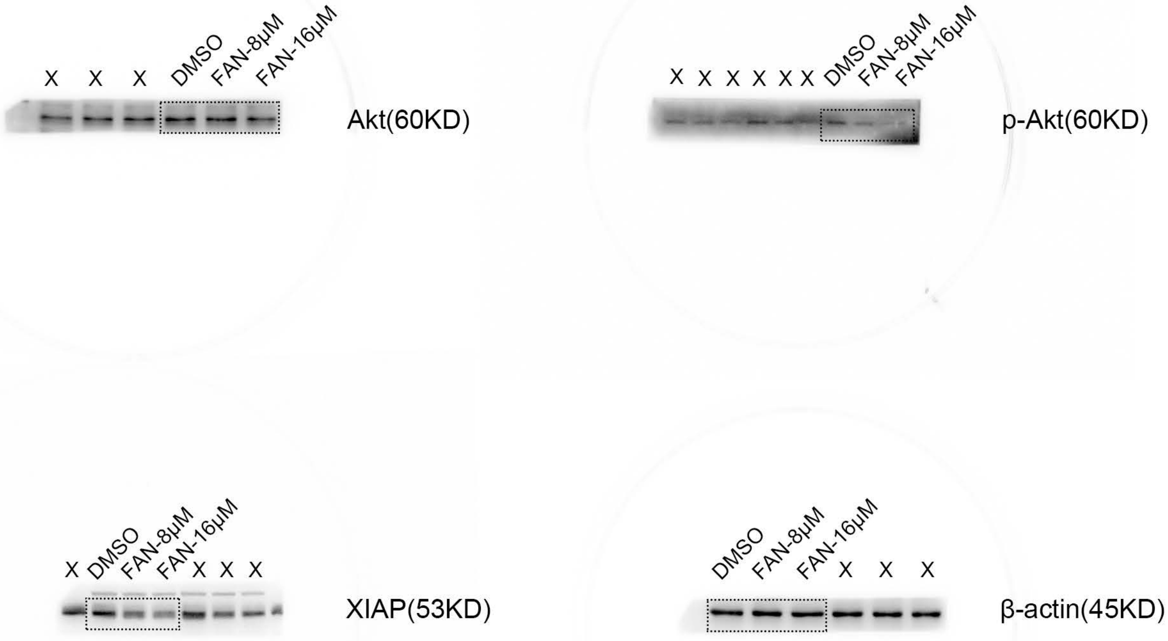
X
X
X
DMSO
FAN-8μM
FAN-16μM
Akt(60KD)
X X X X X X
DMSO
FAN-8μM
FAN-16μM
p-Akt(60KD)
X
DMSO
FAN-8μM
FAN-16μM
X X X
XIAP(53KD)
DMSO
FAN-8μM
FAN-16μM
X X X
β-actin(45KD)

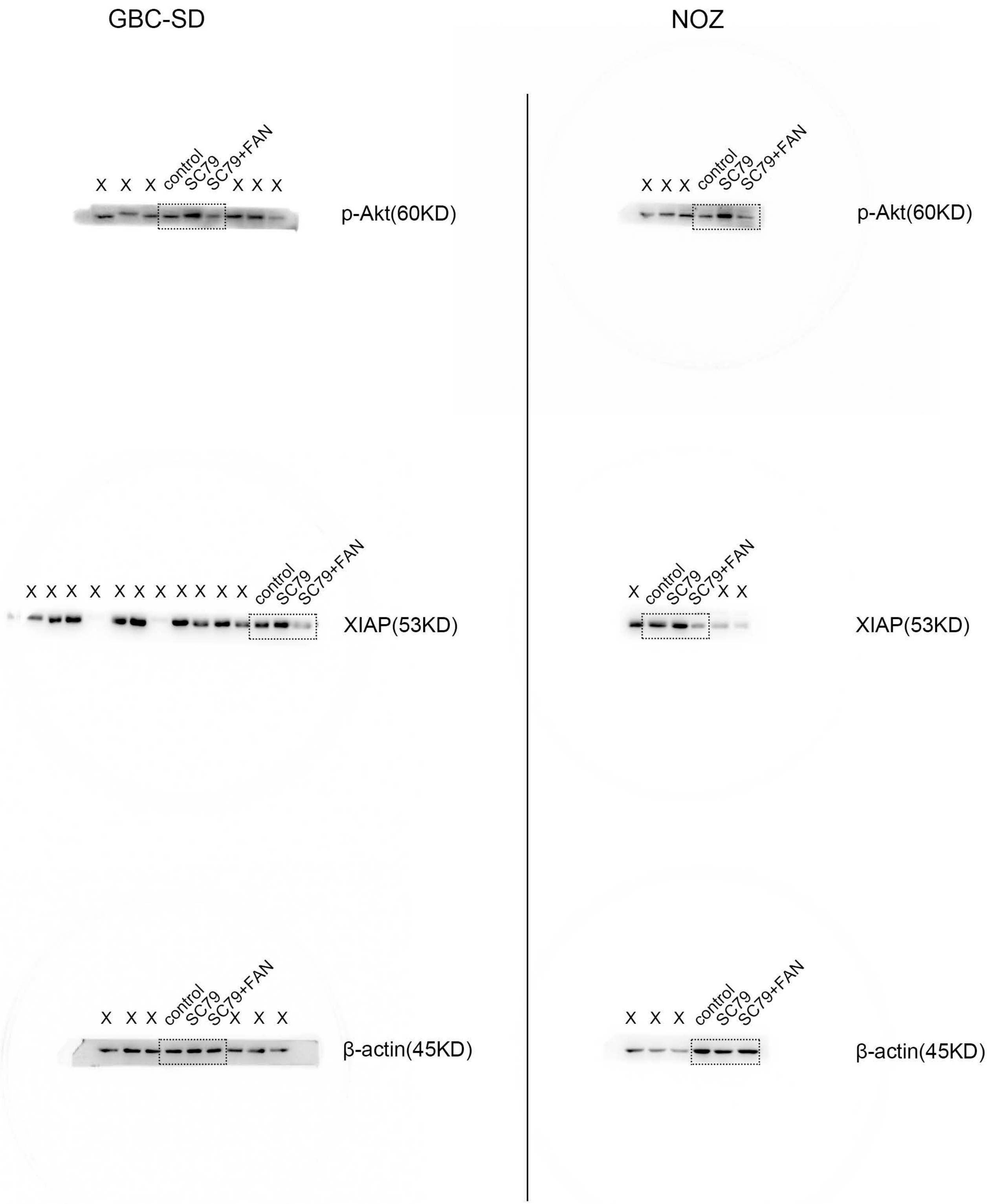

GBC-SD
NOZ
control
SC79
SC79+FAN
p-Akt(60KD)
XIAP(53KD)
β-actin(45KD)
