# Supplementary figures and images for: Fangchinoline induces gallbladder cancer cell apoptosis by suppressing PI3K/Akt/XIAP axis
Source: PLoS One. 2022 Apr 21;17(4):e0266738. doi: 10.1371/journal.pone.0266738 (PMC9022853; doi:10.1371/journal.pone.0266738)

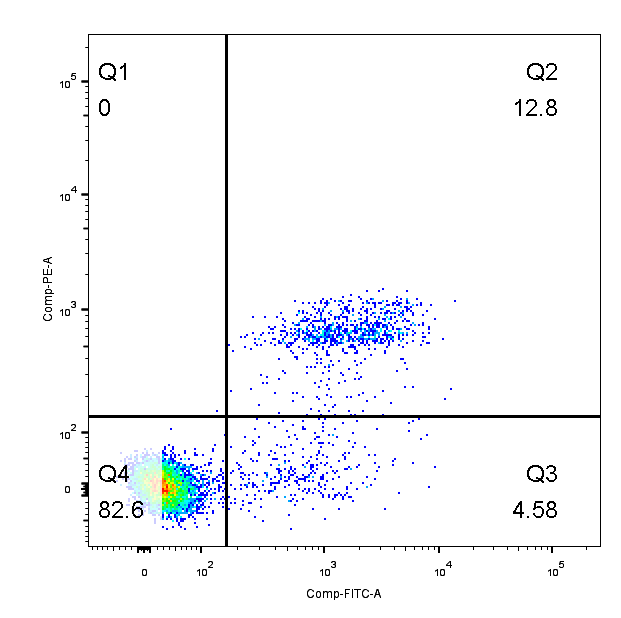

Supplement: S1 File — (ZIP) [file pone.0266738.s001.zip › S1 File/Supporting information/Cell apoptosis/Flow cytometry/GBC-SD(3a╠M).png]

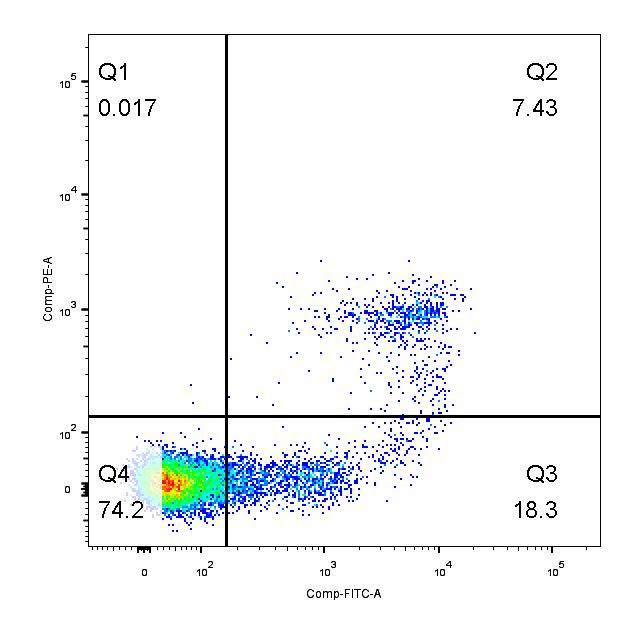

Supplement: S1 File — (ZIP) [file pone.0266738.s001.zip › S1 File/Supporting information/Cell apoptosis/Flow cytometry/GBC-SD(6a╠M).png]

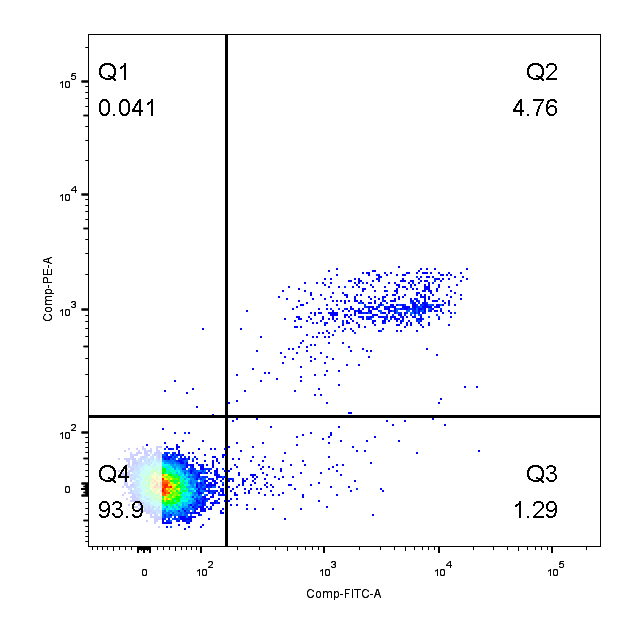

Supplement: S1 File — (ZIP) [file pone.0266738.s001.zip › S1 File/Supporting information/Cell apoptosis/Flow cytometry/GBC-SD(DMSO).png]

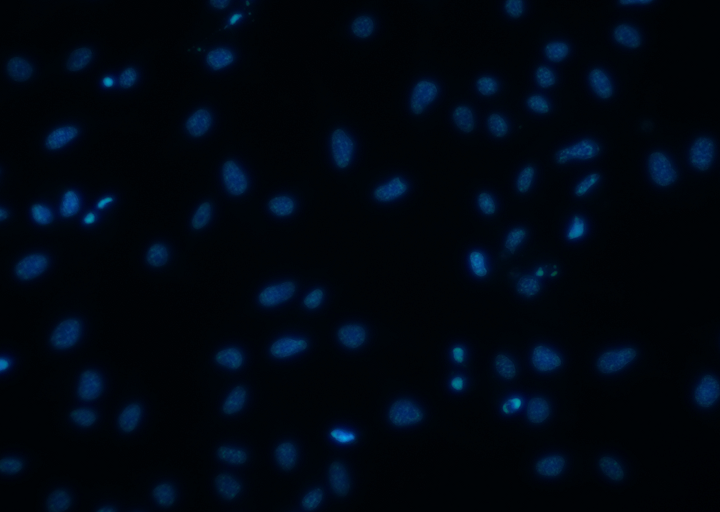

Supplement: S1 File — (ZIP) [file pone.0266738.s001.zip › S1 File/Supporting information/Cell apoptosis/Hoechst staining/GBC-SD/3a╠M(10X).tif]

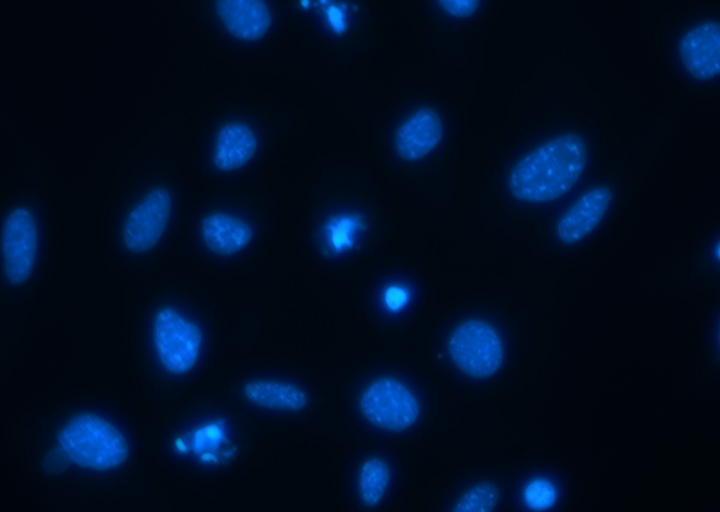

Supplement: S1 File — (ZIP) [file pone.0266738.s001.zip › S1 File/Supporting information/Cell apoptosis/Hoechst staining/GBC-SD/3a╠M(40X).tif]

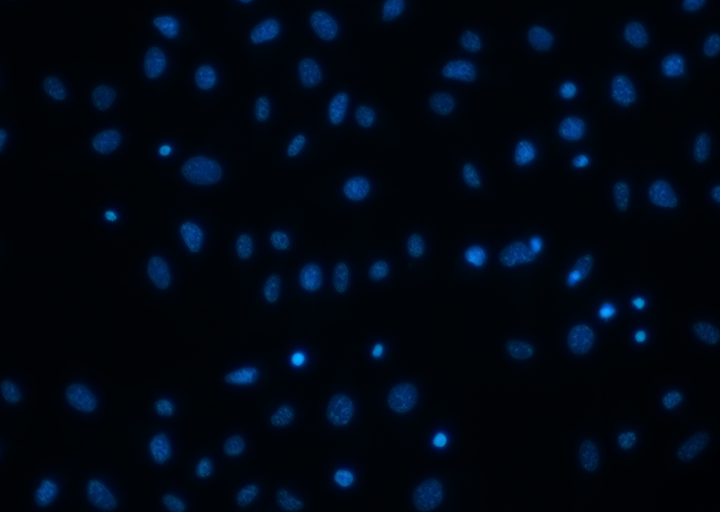

Supplement: S1 File — (ZIP) [file pone.0266738.s001.zip › S1 File/Supporting information/Cell apoptosis/Hoechst staining/GBC-SD/6a╠M(10X).tif]

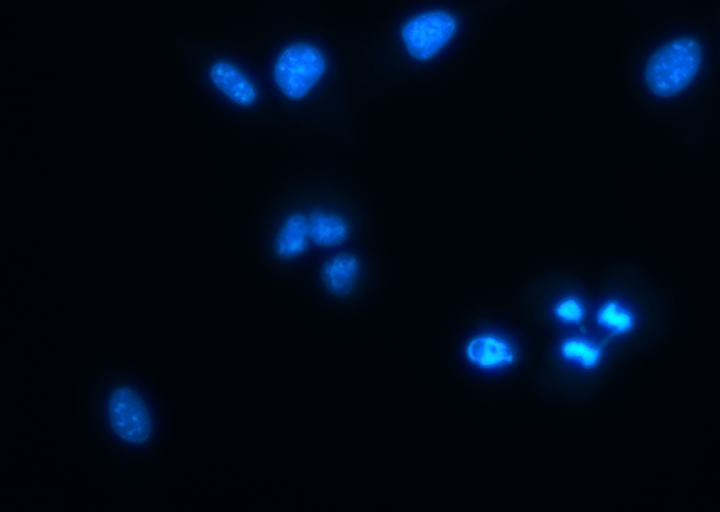

Supplement: S1 File — (ZIP) [file pone.0266738.s001.zip › S1 File/Supporting information/Cell apoptosis/Hoechst staining/GBC-SD/6a╠M(40X).tif]

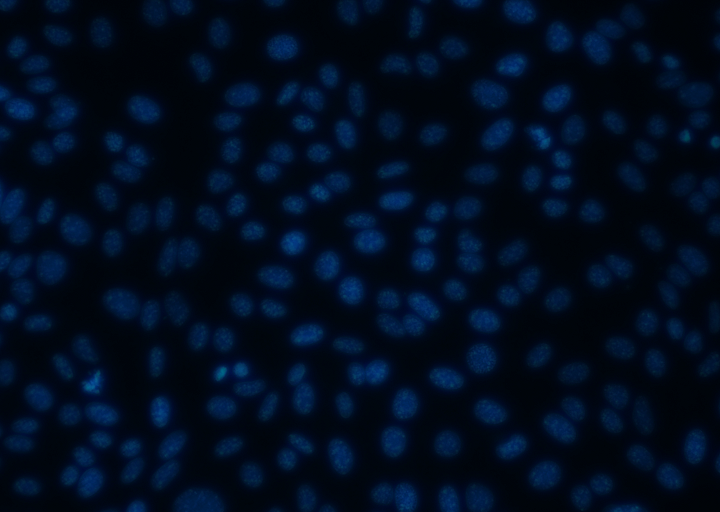

Supplement: S1 File — (ZIP) [file pone.0266738.s001.zip › S1 File/Supporting information/Cell apoptosis/Hoechst staining/GBC-SD/DMSO(10X).tif]

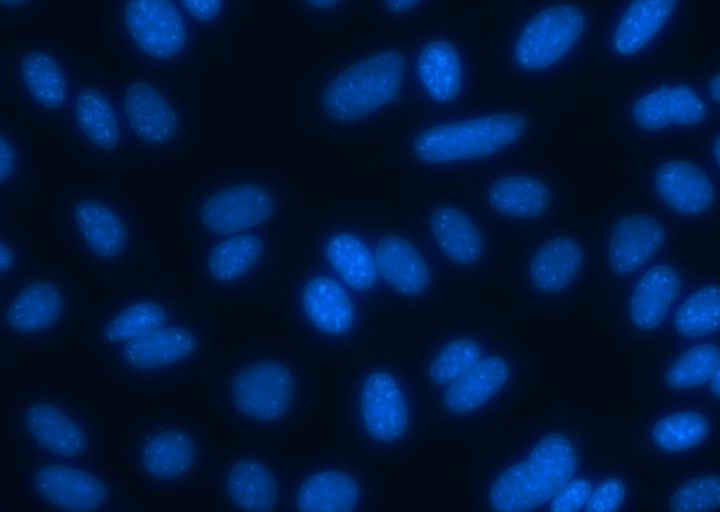

Supplement: S1 File — (ZIP) [file pone.0266738.s001.zip › S1 File/Supporting information/Cell apoptosis/Hoechst staining/GBC-SD/DMSO(40X).tif]

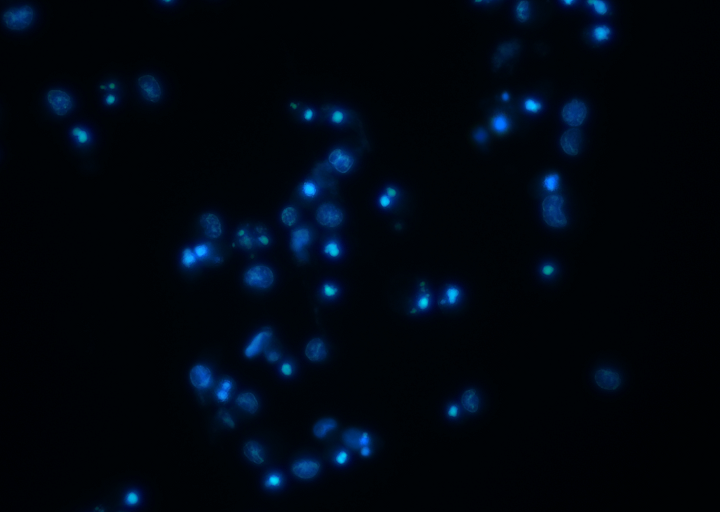

Supplement: S1 File — (ZIP) [file pone.0266738.s001.zip › S1 File/Supporting information/Cell apoptosis/Hoechst staining/NOZ/16a╠M(10X).tif]

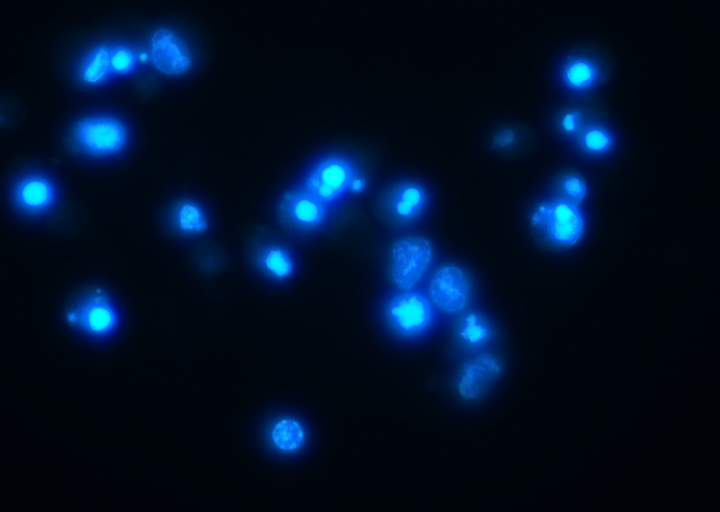

Supplement: S1 File — (ZIP) [file pone.0266738.s001.zip › S1 File/Supporting information/Cell apoptosis/Hoechst staining/NOZ/16a╠M(40X).tif]

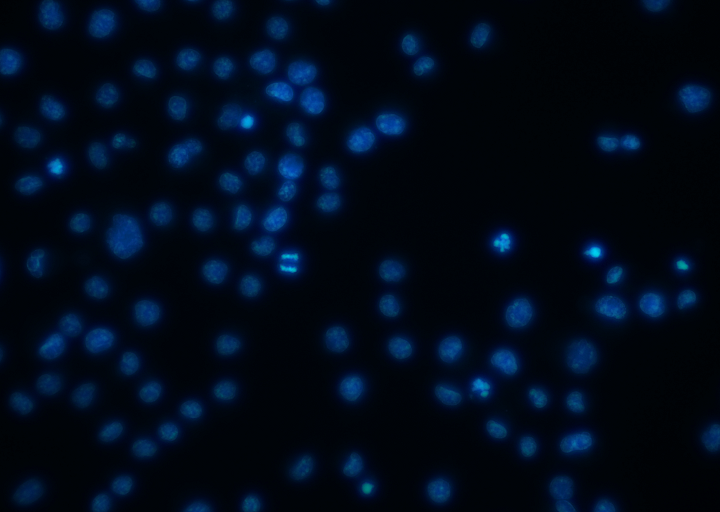

Supplement: S1 File — (ZIP) [file pone.0266738.s001.zip › S1 File/Supporting information/Cell apoptosis/Hoechst staining/NOZ/8a╠M(10X).tif]

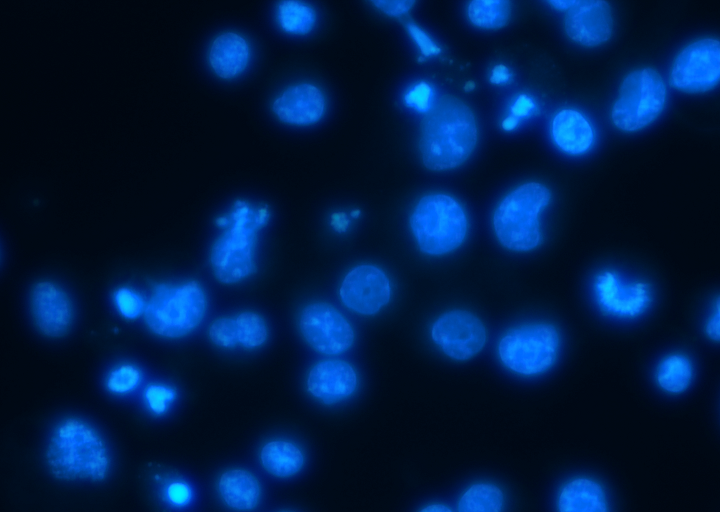

Supplement: S1 File — (ZIP) [file pone.0266738.s001.zip › S1 File/Supporting information/Cell apoptosis/Hoechst staining/NOZ/8a╠M(40X).tif]

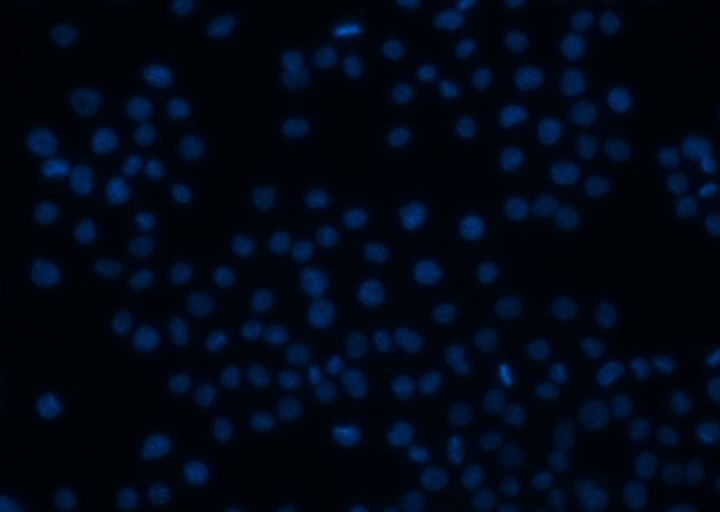

Supplement: S1 File — (ZIP) [file pone.0266738.s001.zip › S1 File/Supporting information/Cell apoptosis/Hoechst staining/NOZ/DMSO(10X).tif]

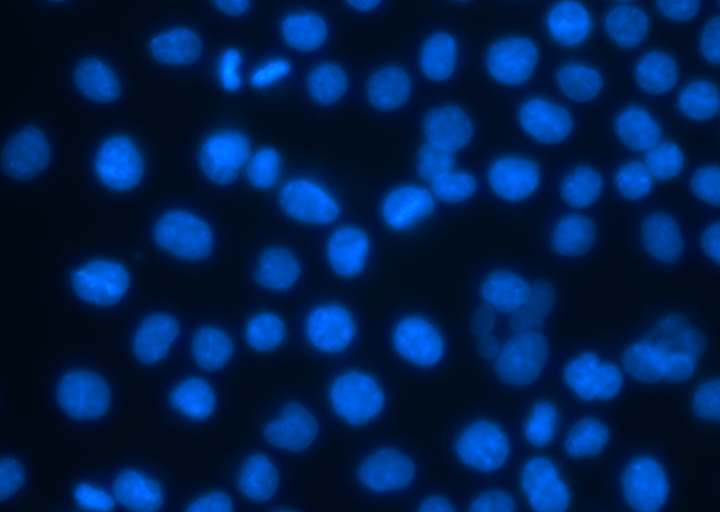

Supplement: S1 File — (ZIP) [file pone.0266738.s001.zip › S1 File/Supporting information/Cell apoptosis/Hoechst staining/NOZ/DMSO(40X).tif]

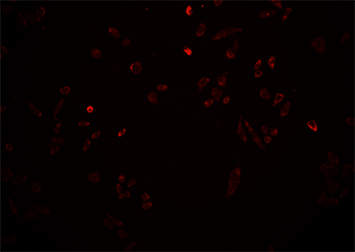

Supplement: S1 File — (ZIP) [file pone.0266738.s001.zip › S1 File/Supporting information/Cell apoptosis/TUNEL staining/GBC-SD(6a╠M) (1).png]

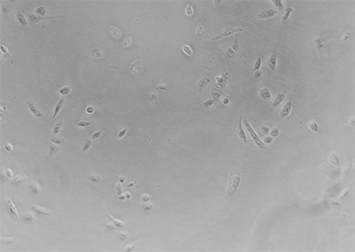

Supplement: S1 File — (ZIP) [file pone.0266738.s001.zip › S1 File/Supporting information/Cell apoptosis/TUNEL staining/GBC-SD(6a╠M) (2).png]

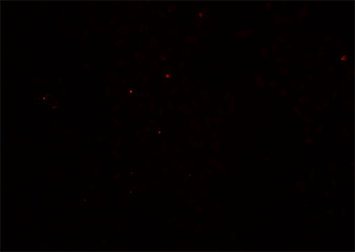

Supplement: S1 File — (ZIP) [file pone.0266738.s001.zip › S1 File/Supporting information/Cell apoptosis/TUNEL staining/GBC-SD(DMSO) (1).png]

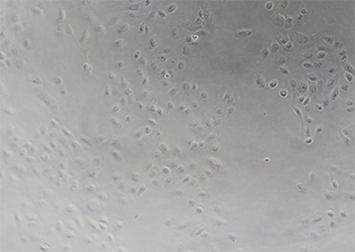

Supplement: S1 File — (ZIP) [file pone.0266738.s001.zip › S1 File/Supporting information/Cell apoptosis/TUNEL staining/GBC-SD(DMSO) (2).png]

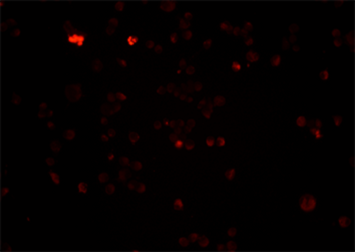

Supplement: S1 File — (ZIP) [file pone.0266738.s001.zip › S1 File/Supporting information/Cell apoptosis/TUNEL staining/NOZ(16a╠M) (1).png]

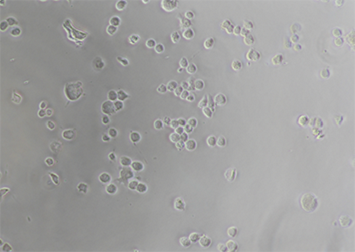

Supplement: S1 File — (ZIP) [file pone.0266738.s001.zip › S1 File/Supporting information/Cell apoptosis/TUNEL staining/NOZ(16a╠M) (2).png]

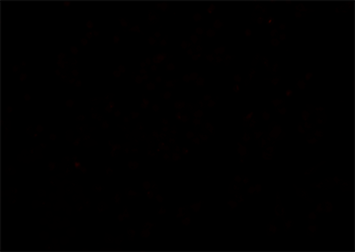

Supplement: S1 File — (ZIP) [file pone.0266738.s001.zip › S1 File/Supporting information/Cell apoptosis/TUNEL staining/NOZ(DMSO) (1).png]

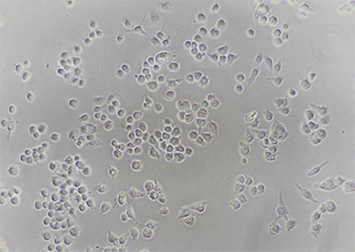

Supplement: S1 File — (ZIP) [file pone.0266738.s001.zip › S1 File/Supporting information/Cell apoptosis/TUNEL staining/NOZ(DMSO) (2).png]

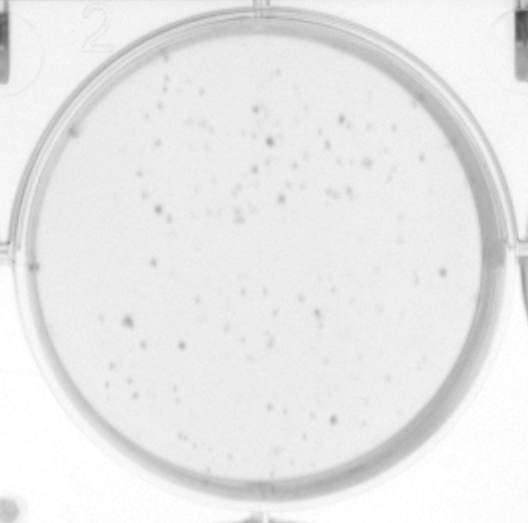

Supplement: S1 File — (ZIP) [file pone.0266738.s001.zip › S1 File/Supporting information/Cell proliferation/Colony formation/GBC-SD(3a╠M).tif]

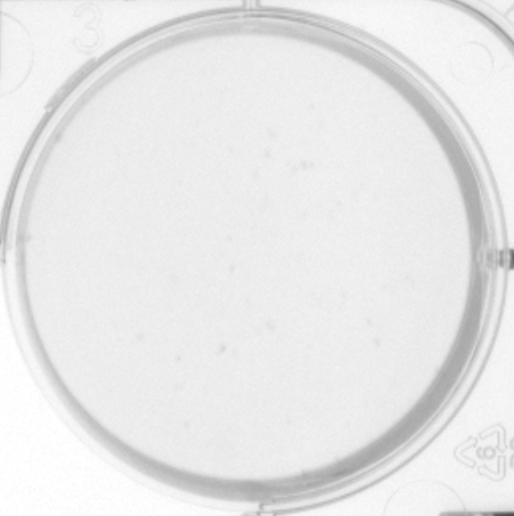

Supplement: S1 File — (ZIP) [file pone.0266738.s001.zip › S1 File/Supporting information/Cell proliferation/Colony formation/GBC-SD(6a╠M).tif]

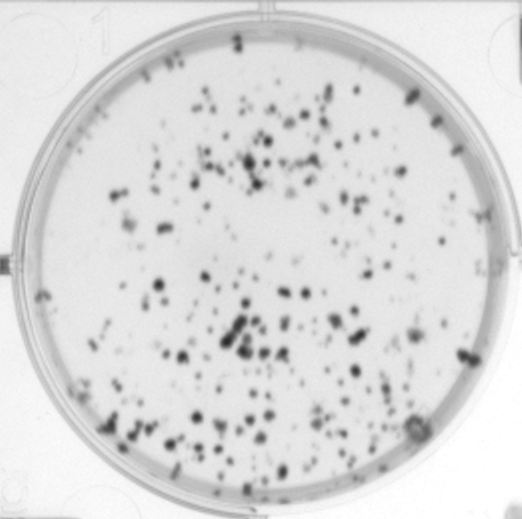

Supplement: S1 File — (ZIP) [file pone.0266738.s001.zip › S1 File/Supporting information/Cell proliferation/Colony formation/GBC-SD(DMSO).tif]

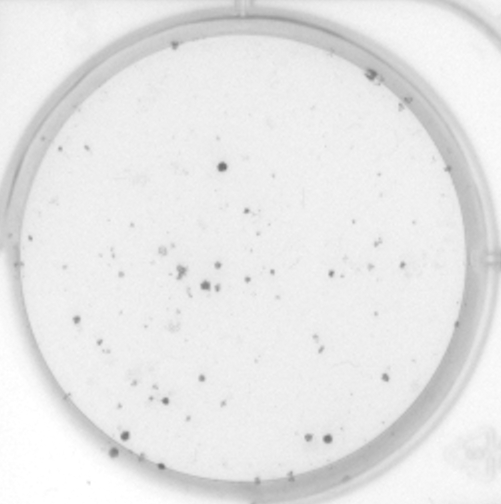

Supplement: S1 File — (ZIP) [file pone.0266738.s001.zip › S1 File/Supporting information/Cell proliferation/Colony formation/NOZ(16a╠M).tif]

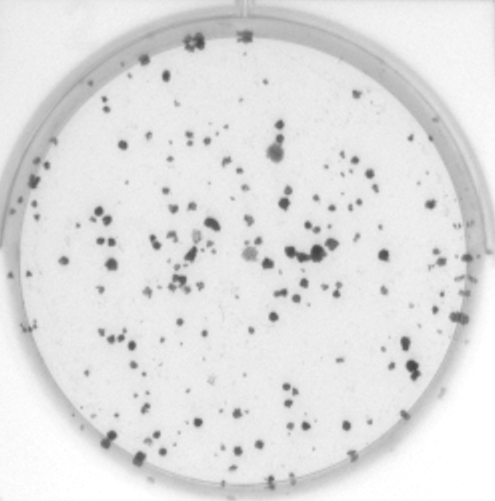

Supplement: S1 File — (ZIP) [file pone.0266738.s001.zip › S1 File/Supporting information/Cell proliferation/Colony formation/NOZ(8a╠M).tif]

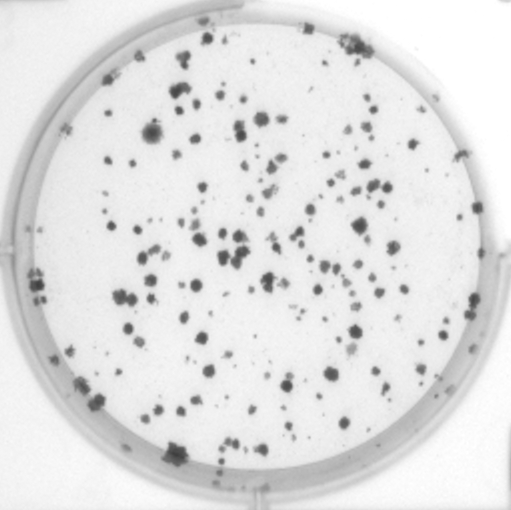

Supplement: S1 File — (ZIP) [file pone.0266738.s001.zip › S1 File/Supporting information/Cell proliferation/Colony formation/NOZ(DMSO).tif]

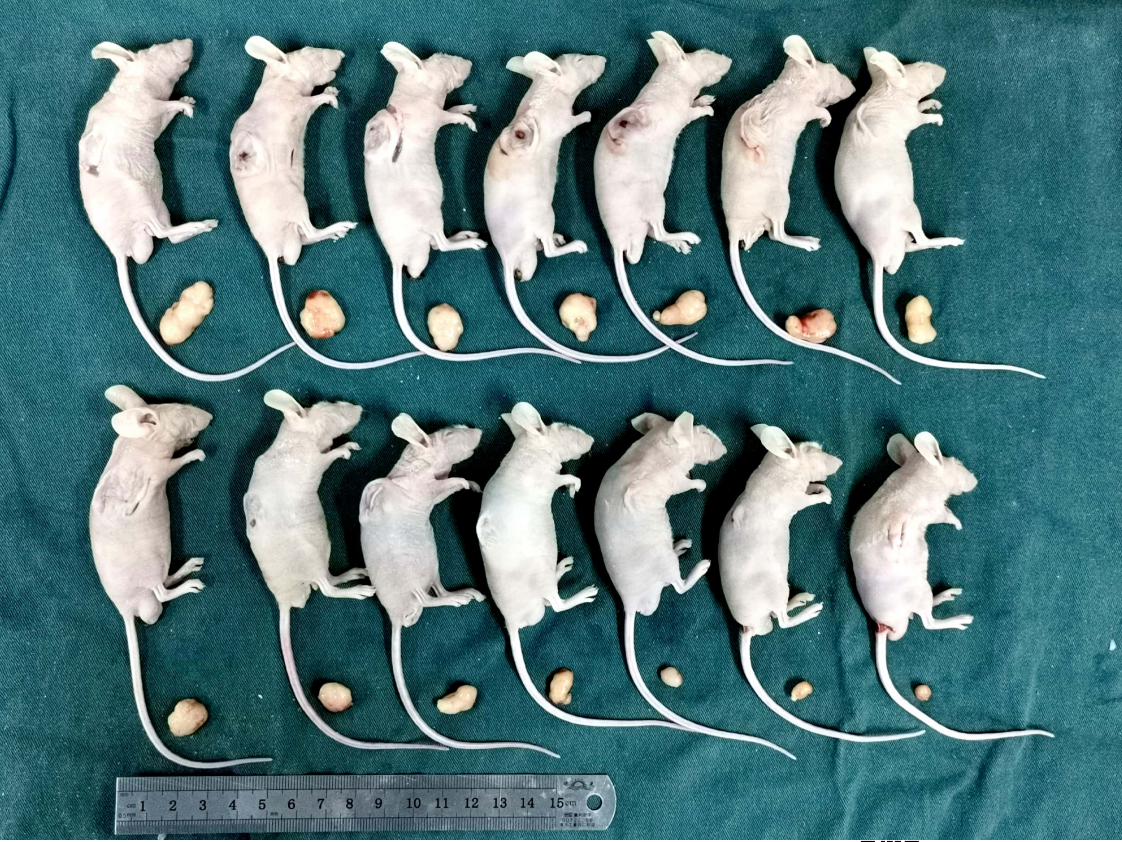

Supplement: S1 File — (ZIP) [file pone.0266738.s001.zip › S1 File/Supporting information/Xenograft tumor/Xenograft tumor.png]
