# Supplementary material for: Fangchinoline induces gallbladder cancer cell apoptosis by suppressing PI3K/Akt/XIAP axis
Source: PLoS One. 2022 Apr 21;17(4):e0266738. doi: 10.1371/journal.pone.0266738 (PMC9022853; doi:10.1371/journal.pone.0266738)
Supplement: Attachment — Submitted filename: Response to Reviewers.doc [file pone.0266738.s002.doc]

Re: Manuscript reference No. : PONE-D-21-34482

Title: Fangchinoline induces gallbladder cancer cell apoptosis by suppressing PI3K/Akt/XIAP axis

PLOS ONE

February 13, 2022

**Responses to the** **comments of** **Reviewer** **#1**

I consider that this article contains important results on the effect of fangchinoline (a bisbenzylisoquinoline alkaloid) wher the authors demonstrated the inhibition of the proliferation and clone formation of GBC cells in a dose-dependent manner and the induction of apoptosis in GBC cells. Authors also showed that fangchinoline acts in vivo by restraining xenograft tumor growth on six-week-old male athymic nude mice.
The document is written correctly and there is a good review of the literature on the subject developed, in addition to meeting the criteria of the journal, so I consider it can be published without problem.

*Response: Thank you for the Reviewer’s positive comments.*

**Responses to the comments of Reviewer #2**

1. The manucript Fangchinoline induces gallbladder cancer cell apoptosis by suppressing PI3K/Akt/XIAP axis lacks the selection of this alkaloid as an inhibitor of PI3K/Akt/XIAP? why not PI3K/Akt/mTOR?

*Response: As the comments of Reviewer, we have examined the PI3K/Akt/mTOR axis and provided the results in the revised manuscript (Fig3). Unfortunately, the difference of p-mTOR was not significant with or without Fangchinoline treatment (Fig3). On the other hand, the relationship between XIAP and Akt had been further confirmed by an Akt activator, SC79 (Fig3).*

2. Also, the rationality is required to show the anti-cancer activity of Fangchinoline. The author should use positive control in all the assays.

*Response: The suggestion from the reviewer is helpful. However, the aim of our study is to determine whether Fangchinoline effectively inhibits GBC proliferation in vivo and in vitro, rather than to compare it with other anti-cancer drugs. Similar research strategy has been used by abundant studies.*

3. Fangchinoline suppresses the proliferation, invasion and tumorigenesis of human osteosarcoma cells through the inhibition of PI3K and downstream signaling pathways is reported IJMM 2017. So better the author compare some results with the published mode of action of this alkaloid and resubmit.

*Response: As the comments of Reviewer, the comparison of relevant conclusions has been added to our revised manuscript.*

**Responses to the comments of** **Editor**

1. Please ensure that your manuscript meets PLOS ONE's style requirements, including those for file naming.

*Response: As the comments of Editor, we have mad revisions in our revised manuscript, including style and file naming.*

2. To comply with PLOS ONE submissions requirements, in your Methods section, please provide additional information on the animal research and ensure you have included details on (1) methods of sacrifice, (2) methods of anesthesia and/or analgesia, (3) efforts to alleviate suffering, (4) number of animals used in the study.

*Response: As the comments of Editor, the additional information on the animal research has been added into the section of Xenograft formations assays.*

3. Thank you for stating the following in the Acknowledgments Section of your manuscript:

“The work was sponsored by Zhejiang Provincial Natural Science Foundation of China under grant no. LY19H160016, National Natural Science Foundation of China (NSFC) no. 81602044, Zhejiang Provincial Medical and Health Science and Technology Project under grant no. 2019ZD057.”

Please note that funding information should not appear in the Acknowledgments section or other areas of your manuscript. We will only publish funding information present in the Funding Statement section of the online submission form.

Please remove any funding-related text from the manuscript and let us know how you would like to update your Funding Statement. Currently, your Funding Statement reads as follows:

“This work was supported by National Natural Science Foundation of China (NSFC) no. 81602044 and Zhejiang Provincial Natural Science Foundation of China under grant no. LY19H160016. Dr. Jianhua Yu received these funding and was responsible for the decision to publish this manuscript.

This work was also supported by Zhejiang Provincial Medical and Health Science and Technology Project under grant no. 2019ZD057. Dr. Baochun Lu received the funding and was responsible for data collection of this manuscript.”

Please include your amended statements within your cover letter; we will change the online submission form on your behalf.

*Response: As the comments of Editor, the funding-related text in manuscript has been removed. Meanwhile, we have updated the funding information as the comments of Editor:*

*“This work was supported by Zhejiang Provincial Natural Science Foundation of China under grant no. LY22H160008, LY19H160016, National Natural Science Foundation of China (NSFC) no. 81602044, Zhejiang Provincial Medical and Health Science and Technology Project under grant no. 2022RC080 and 2020RC127. Dr. Jianhua Yu received these funding and was responsible for the decision to publish this manuscript.*

*This work was also supported by Zhejiang Provincial Natural Science Foundation of China under grant no. LBY22H030001 and Zhejiang Provincial Medical and Health Science and Technology Project under grant no. 2019ZD057. Dr. Baochun Lu received the funding and was responsible for data collection of this manuscript.”*

*Thanks to the Editors for changing the online submission form on our behalf.*

4. In your Data Availability statement, you have not specified where the minimal data set underlying the results described in your manuscript can be found.

*Response: As the comments of Editor, the minimal data set underlying results has been added into our supplementary materials. All relevant data now are within the revised manuscript and its Supporting information files.*

5. PLOS ONE now requires that authors provide the original uncropped and unadjusted images underlying all blot or gel results reported in a submission’s figures or Supporting Information files.

*Response: As the comments of Editor, the original uncropped and unadjusted images underlying all blot or gel results have been uploaded and named ‘S1_raw_images’.*

6. Please review your reference list to ensure that it is complete and correct. If you have cited papers that have been retracted, please include the rationale for doing so in the manuscript text, or remove these references and replace them with relevant current references. Any changes to the reference list should be mentioned in the rebuttal letter that accompanies your revised manuscript. If you need to cite a retracted article, indicate the article’s retracted status in the References list and also include a citation and full reference for the retraction notice.

*Response: We have reviewed our reference list as the Reviewer suggested and there are no papers have been retracted. It’s worth noting that a new papers was cited in our Discussion section.*

***Reference***

1.	Huang C-Y, Chang Y-J, Wei P-L, Hung C-S, Wang W. Methyl gallate, gallic acid-derived compound, inhibit cell proliferation through increasing ROS production and apoptosis in hepatocellular carcinoma cells. PLoS One. 2021;16(3):e0248521. doi: 10.1371/journal.pone.0248521. PubMed PMID: 33725002.

2.	Somasagara RR, Hegde M, Chiruvella KK, Musini A, Choudhary B, Raghavan SC. Extracts of strawberry fruits induce intrinsic pathway of apoptosis in breast cancer cells and inhibits tumor progression in mice. PLoS One. 2012;7(10):e47021. doi: 10.1371/journal.pone.0047021. PubMed PMID: 23071702.

3.	Singh T, Sharma SD, Katiyar SK. Grape proanthocyanidins induce apoptosis by loss of mitochondrial membrane potential of human non-small cell lung cancer cells in vitro and in vivo. PLoS One. 2011;6(11):e27444. doi: 10.1371/journal.pone.0027444. PubMed PMID: 22087318.

4.	Wu H, Chen L, Zhu F, Han X, Sun L, Chen K. The Cytotoxicity Effect of Resveratrol: Cell Cycle Arrest and Induced Apoptosis of Breast Cancer 4T1 Cells. Toxins (Basel). 2019;11(12). doi: 10.3390/toxins11120731. PubMed PMID: 31847250.

5.	Kong Y, Chen G, Xu Z, Yang G, Li B, Wu X, et al. Pterostilbene induces apoptosis and cell cycle arrest in diffuse large B-cell lymphoma cells. Sci Rep. 2016;6:37417. doi: 10.1038/srep37417. PubMed PMID: 27869173.

6.	Li X, Yang Z, Han W, Lu X, Jin S, Yang W, et al. Fangchinoline suppresses the proliferation, invasion and tumorigenesis of human osteosarcoma cells through the inhibition of PI3K and downstream signaling pathways. Int J Mol Med. 2017;40(2):311-8. doi: 10.3892/ijmm.2017.3013. PubMed PMID: 28586029.
